# The Effect of Long-Term Non-Invasive Ventilation on Tracheostomy-Free Survival and Hospitalizations in Types 2 and 3 Spinal Muscular Atrophy Patients

**DOI:** 10.3390/jcm14228171

**Published:** 2025-11-18

**Authors:** Andrea Vianello, Gabriella Guarnieri, Leonardo Bertagna De Marchi, Beatrice Molena, Giovanna Arcaro, Giuliana Capece, Elena Sogus, Federico Lionello, Elena Pegoraro

**Affiliations:** 1 Department of Cardiac, Thoracic, Vascular Sciences and Public Health, University of Padova, Via Giustiniani 2, 35128 Padova, Italy; gabriella.guarnieri@unipd.it (G.G.); leonardo.bertagnademarchi@aopd.veneto.it (L.B.D.M.); beatrice.molena@unipd.it (B.M.); giovanna.arcaro@aopd.veneto.it (G.A.); federico.lionello@aopd.veneto.it (F.L.); 2 Fisiopatologia Respiratoria, Ospedale-Università di Padova, Via Giustiniani 2, 35128 Padova, Italy; 3 Department of Neuroscience, University of Padova, Via Giustiniani 2, 35128 Padova, Italy; giuliana.capece@aopd.veneto.it (G.C.); elena.sogus@aopd.veneto.it (E.S.); elena.pegoraro@unipd.it (E.P.)

**Keywords:** long-term non-invasive ventilation, spinal muscular atrophy, tracheostomy, hospitalization

## Abstract

**Background:** The impact of Long-Term Non-Invasive Ventilation (LT-NIV) on patient-relevant outcomes has yet to be clearly established in types 2 and 3 Spinal Muscular Atrophy (SMA). **Objectives:** The current study aimed to assess LT-NIV effect(s) on tracheostomy-free survival and the need for hospitalization. Its secondary aim was to identify patient characteristics that can be considered risk factors for an unsatisfactory response to treatment. **Design:** This study is a retrospective analysis of long-term outcomes in types 2 and 3 SMA patients receiving home LT-NIV. **Methods:** *Patients and interventions*: Eighteen types 2 and 3 SMA patients who were receiving home LT-NIV between 1 January 1990 and 31 March 2025 were included. *Primary study endpoint*: The endpoint comprised a composite of tracheostomy-free survival time and hospitalization rate. **Results:** Twelve patients (66.7%) had a positive response to LT-NIV (group A); the response was unsatisfactory in the remaining 6 (33.3%) (group B). Tracheostomy-free survival time was significantly reduced in the latter compared to the former [66 (range: 2–172) vs. 280 (range: 67–407) months; *p* = 0.010] and the hospitalization rate was significantly higher [0.35 (range: 0.06–1.44) vs. 0.07 (range: 0.00–0.18) episodes/yr; *p* = 0.007]. A correlation was found between an unsatisfactory response to LT-NIV and treatment initiation following an episode of acute respiratory failure (OR: 7.90; 95% CI, 0.99–123.2; *p* = 0.051). **Conclusions:** LT-NIV has a positive impact on tracheostomy-free survival and hospitalizations in types 2 and 3 SMA patients. The risk of an unsatisfactory response appears to be higher when the treatment is initiated in clinically unstable patients.

## 1. Introduction

Spinal Muscular Atrophy (SMA) is a rare autosomal recessive disorder that causes progressive proximal muscle weakness and paralysis due to the degeneration of alpha motor neurons in the spinal cord and brainstem caused by biallelic variants in the survival motor neuron 1 (SMN1) gene on the long arm of chromosome 5q [1]. Before disease-modifying treatment became available, SMA was traditionally classified into five main types: 0, 1, 2, 3, and 4. The classification was historically based on both the age at onset and the severity of the symptoms of motor control dysfunction. Classically, type 2 intermediate SMA manifests between 6 and 18 months of age in patients who generally achieve the ability to sit (“sitters”) but not to walk independently [2]. Children with type 3 SMA typically reach all major motor milestones and achieve independent ambulation (“walkers”) but present proximal weakness, mostly in the legs at some time between 18 months and 18 years of age. Depending on whether the limb weakness presents before or after 3 years of age, these patients can be sub-classified as having type 3a or type 3b SMA [3].

Respiratory impairment is the most frequent non-neurologic complication in type 2 SMA patients and the major cause of morbidity and mortality [4,5]. Indeed, impaired respiratory muscle function may result in the following: (a) the development of a bell-shaped chest with sternal depression due to an imbalance between maintained diaphragmatic contractility and reduced intercostal muscle contractility; (b) impaired cough ability resulting in ineffective removal of airway secretions and airway mucous, frequently complicated by pneumonia, atelectasis, and, ultimately, acute respiratory failure (ARF); and (c) sleep-disordered breathing (SDB), leading to nocturnal hypoventilation and worsening of gas exchange [6,7]. The natural history of pulmonary compromise invariably leads to chronic respiratory failure (CRF) [6]. Of note, type 3a SMA patients may show ventilatory patterns similar to those of patients affected by type 2 SMA [8].

Non-invasive ventilation (NIV), a form of ventilatory support without airway invasion, is essential to the respiratory care of type 2 SMA children: indeed, long-term nighttime use of NIV (LT-NIV) has been found to have a beneficial effect on a variety of physiological parameters, including thoraco-abdominal coordination, arterial blood gases (ABG), and sleep quality. Finally, it seems to stabilize lung and respiratory muscle function [9,10,11]. Regular LT-NIV use also seems to ensure relevant benefits in a significant proportion (5–33%) of type 3 SMA patients [4,12]. Given these encouraging findings, leading experts recommended LT-NIV for all type 2 and 3 SMA patients reporting symptoms of nocturnal hypoventilation (i.e., poor sleep quality, daytime sleepiness, difficulty concentrating, morning headaches, anorexia, nocturnal sweating, or waking with panic sensation), using an individualized approach based on the subject’s needs and preferences [13,14]. Although the reports of beneficial physiological effects seem to support the hypothesis that LT-NIV improves patient-relevant outcomes, such as symptoms of nocturnal hypoventilation, physical function/functional status and/or Quality of Life (QoL) [15], only a few inconclusive studies have reported on its impact on patient-relevant endpoints, in particular the need for a tracheostomy and/or repeated hospitalizations [9,16,17,18]. Indeed, the placement of a tracheostomy tube can significantly worsen patients’ QoL and dramatically augment the families’ burden of care [19]. Repeated hospitalizations can likewise adversely affect the independence and QoL of patients and their families [20]. In view of these considerations, this study set out to evaluate and analyze the medical records of types 2 and 3 SMA patients receiving LT-NIV prescribed by the outpatient clinic of a pulmonary division of a tertiary hospital over a 35-year period. This study specifically aimed to assess the effect of LT-NIV on the patients’ tracheostomy-free survival time and the need for hospitalization and to identify those clinical and laboratory characteristics which could be considered risk factors for an unsatisfactory response to the ventilatory support therapy.

## 2. Methods

This observational retrospective single-center study was conducted in a tertiary teaching hospital located in Northeast Italy. All of the study participants were asked to sign informed consent forms agreeing to the use of their de-identified clinical data for research, analysis, and reporting purposes. Those younger than 18 years of age reached this decision in accordance with their parents. In accordance with regional regulations (No. 2772-30 December 2022), the need for ethical approval was waived by the facility’s Institutional Review Committee given that this study was retrospective in nature and all the interventions prescribed were part of standard protocols. This study was carried out in accordance with the Declaration of Helsinki of 1975. STROBE guidelines for reporting cohort studies were consulted when preparing the manuscript.

### 2.1. Participants

All of the types 2 and 3 SMA patients who were prescribed home LT-NIV after 1 January 1990 (the date that the home care services for ventilator-dependent patients was activated in our geographic area) and were followed up by the medical staff of the outpatient clinic of the Respiratory Pathophysiology Division of the University of Padua Medical Center up until the end of the study period (31 March 2025) or death were considered eligible for our retrospective study. LT-NIV was defined as the regular daily use of NIV at home for a minimum of 4 h per night for ≥3 consecutive months [21,22]. With the exception of 4 persons who declined undergoing diagnostic testing, all of the patients had a genetically confirmed diagnosis of SMA based on molecular genetic testing identifying mutations in the SMN1 gene. Clinically, the diagnosis of SMA was established in accordance with the criteria of the Standard of Care in Spinal Muscular Atrophy (SMA SOC) Consensus statement first published in 2007 [14] and subsequently updated [23].

All of the patients’ medical records were reviewed for demographic, clinical, and pulmonary function information, and the following data were collected and recorded:

(A) At the time the decision was made to initiate NIV:

The patient’s age, gender, body mass index (BMI), and smoking habit; his or her Revised Upper Limb Module (RULM) score; if the patient had undergone a spinal fusion surgery; if he or she had been placed with a percutaneous gastrostomy (PEG) tube; and if NIV was initiated as an elective therapy or following an ARF episode. RULM is a scale designed to specifically assess the upper limb function in SMA individuals [24]. The patient’s Forced Vital Capacity (FVC) values from pulmonary function tests (PFTs) that were carried out in cooperative patients within a six-month period preceding LT-NIV initiation were also recorded. Moreover, the severity of scoliosis was evaluated by calculating the Cobb angle at spine X-ray, with an angle > 40 degrees considered as severe scoliosis [25].

(B) During the follow-up period:
  - The reasons why it had become necessary to discontinue LT-NIV, classified as one of the following: (a) death due to cardio-respiratory acute illness; (b) tracheostomy due to cardio-respiratory acute illness, NIV complications, or full-time ventilator dependence; or (c) lack of motivation.
  - If complications associated with NIV use had arisen. Complications were classified as follows: (a) major complications, including those that are potentially life-threatening or lead to the need for intubation and/or tracheostomy; or (b) minor complications, defined as mild or transient medical problems related to features specific to NIV, such as the interface or airflow [26].
  - If there had been any hospitalizations (in our own or other hospitals) linked to respiratory problems. The conditions associated with hospitalization/s due to respiratory complications were classified as upper respiratory tract infection (URTI), pneumonia, aspiration pneumonia, pneumothorax, pulmonary thromboembolism, and abuse of sedatives. The diagnosis of URTI was based on the presence of one or more of the following signs or symptoms: fever, throat irritation or sore throat, and hoarseness [27]. The diagnosis of pneumonia was made in accordance with current guidelines [28].
  - If a therapy that enhanced the production of SMN protein had been prescribed. In our case, Nusinersen (Spinraza, Biogen, Cambridge, MA, USA) and/or Risdiplam (Evrysdi, Genentech/Roche, South San Francisco, CA, USA) were considered.
  - The patient’s FVC values from last PFT carried out before tracheostomy tube was placed, the study period came to an end, or the patient died.

### 2.2. Interventions

#### 2.2.1. Non-Invasive Ventilation

LT-NIV was initiated either as an elective therapy as part of a decision-sharing approach or following an ARF episode. In line with published guidelines [29], elective LT-NIV was initiated in patients with chronic hypercapnic respiratory failure and/or sleep-disordered breathing (SDB). The indications for home LT-NIV included the following: (a) daytime PaCO_2_ > 45 mmHg; and/or (b) symptoms of nocturnal hypoventilation (morning headache, daytime hypersomnolence, sleep disturbed by frequent awakenings) associated with nocturnal oximetry showing SpO_2_ levels < 88% for at least five consecutive minutes. The decision to initiate elective NIV, which was made during an inpatient assessment, was based upon the results of an overnight transcutaneous capnometry and/or of sequential arterial blood gas (ABG) tests. Post-acute LT-NIV was prescribed following an episode of ARF requiring any type of ventilatory support. Contraindications to LT-NIV included the following: (a) severely impaired swallowing leading to chronic aspiration and repeated pneumonia; and (b) ineffective airway clearance despite the use of non-invasive manual or mechanical expiratory aids.

Between 1990 and 2000, lightweight, elastic, custom-made nasal masks were utilized as the interface for NIV provision. After that date, commercially available silicone nasal masks have been used. Family care-givers were provided information, instruction, and training on NIV use by the respiratory therapists of our division. Care-givers’ preparation, which was usually organized over a 3-day period, consisted in basic life support instruction and in training on how to use the NIV and oximetry devices and to identify a respiratory emergency. The patients were instructed to use their NIV device throughout the entire night during sleep and to extend its use during daytime as the disease progressed. The duration of NIV use over a 24 h period was categorized as nocturnal (use up to 8 h/24), intermediate (use between 8 and 16 h/24), or life support (use ≥ 16 h/24) [21].

#### 2.2.2. Other Interventions

Assisted coughing techniques (ACTs): Depending on the patient’s clinical status and level of cooperation, manually and/or mechanically assisted coughing was provided by family care-givers to improve his/her ability to clear secretions in the event of acute exacerbation. Beginning in 2000, a mechanical Insufflation–Exsufflation (MI-E) device was made available. Its use was indicated for individuals with chest wall stiffness (i.e., severe thoracic deformity or obesity) and for those subjects who were unable to fully perform deep insufflation. The device’s positive and negative pressures and the timing were independently adjusted depending on its efficacy and patient tolerance; the pressures were generally set between +30 and −40 cm H_2_O.

District nurse’s home visits: A service of district nurses visited the patients on a quarterly basis. The nurse assessed the patient’s adherence and response to treatment and set up an appointment with the pulmonologist if the patient’s clinical progress was considered unsatisfactory.

Annual influenza and pneumococcal immunizations were strongly recommended for all the patients.

### 2.3. Study Endpoint and Statistical Analysis

The primary study endpoint was a composite of tracheostomy-free survival time and hospitalization rate. According to the results of other studies examining LT-NIV in children/adolescents suffering from various kinds of neuromuscular disorders [16,17], our patients were divided into 2 groups depending on their LT-NIV response. The first group (group A) was made up of those individuals whose response to treatment was considered successful, as defined by a tracheostomy-free survival time ≥ 5 yrs and hospitalization rate < 1/yr during the study period. The second group (group B) was made up of those subjects whose response to NIV treatment was considered unsuccessful, as defined by a tracheostomy-free survival < 5 yrs and/or hospitalization rate ≥ 1/yr for at least 5 consecutive years. Tracheostomy-free survival was defined as the number of years between the time LT-NIV was initiated and the time one of the following occurred: a tracheostomy tube was placed, the study period came to an end, or the patient died. The hospitalization rate was defined as the number of hospitalizations per year calculated for every year from the time LT-NIV was initiated to the time one of the following occurred: a tracheostomy tube was placed, the study period came to an end, or the patient died.

The patients’ outcomes, as deduced from their clinical status parameters, were analyzed. The results are expressed, as appropriate, as mean or median values, ranges, or percentages. The continuous variables were compared, depending on the normality of the distributions, using the Student’s *t* test or the Mann–Whitney U test. The categorical variables were compared, as appropriate, using the Chi-squared test or Fisher’s exact test. Variables potentially useful in predicting LT-NIV failure were analyzed using the exact logistic regression model, as this procedure can adequately estimate a binary response variable even for a small sample size [30]. The predictors of interest included all the data recorded in the charts. All the variables with *p*-values of ≤0.1 in univariate analysis were used as independent variables in multivariate analysis. Exact Odds Ratios (ORs) and 95% CI were reported for significant predictors using multivariate logistic analyses. The tracheostomy-free survival time following LT-NIV initiation was calculated using the Kaplan–Meier method; the log-rank test was used to compare the survival curves of the two groups. The patients who were still alive at the end of the study period were censored on 31 March 2025. The patients who were switched to tracheostomy ventilation (TV) were censored at the time NIV was discontinued. A bilateral *p*-value < 0.05 was considered statistically significant for all the comparisons. All of the statistical calculations were carried out using version 2.3 Jamovi 2022 software (www.jamovi.org). 

## 3. Results

During the study period (1 January 1990–31 March 2025), twenty types 2 and 3 SMA patients attending our outpatient clinic were prescribed home LT-NIV. Two were not eligible to participate because some of their clinical records were missing. In the end, 18 were considered eligible to participate in our retrospective study. Ten patients (55.6%) were affected with type 2 SMA and eight (44.4%) with type 3 SMA. The median duration of the follow-up period was 194 (range: 25–422) months. Twelve patients (66.7%) responded well to LT-NIV (group A); the response of the other six (33.3%) (group B) was unsatisfactory. Three out of the six group B patients required a tracheostomy quite soon after LT-NIV was initiated [14 (range: 3–27) months]. In all three cases, the tracheostomy was necessary because of acute cardio-respiratory failure consequent to community-acquired pneumonia requiring endotracheal intubation (ETI) complicated by difficult weaning. The remaining three required frequent hospital admissions due to acute exacerbation [hospitalization rate: 1 (range: 1–1.2) episode/yr]. Four patients experienced minor complications linked to NIV treatment. Two presented facial skin breakdown over the nasal bridge requiring protective dressings or a transition to a full-face mask. Although one child initially reported finding the mask uncomfortable due to nasal congestion, no change in treatment was ultimately required. Another patient experienced acute gastric dilatation with nausea and gastro-esophageal reflux.

The patients’ anthropometric, clinical, pulmonary function, and blood gas data at the time LT-NIV was initiated are outlined in Table 1.

A trend toward higher PaCO_2_ values was noted in the group B patients [43.0 (range: 38.0–70.0) vs. 38 (range: 32.9–41.5) mmHg; *p* = 0.088]. Univariate analysis uncovered that gender had no significant effect on the primary study endpoint, with an HR of 2.92 for males (95% CI, 0.54–15.70; *p* = 0.211). Data on the SMN-enhancing treatment prescribed to the patients in the two groups are outlined and compared in Table 2.

The number of patients receiving Risdiplam was higher, although not significantly, in group A with respect to the group B patients (6/12 vs. 0/6; *p* = 0.114). Data on initiation and outcomes of LT-NIV are outlined in Table 3.

**Figure 1:**
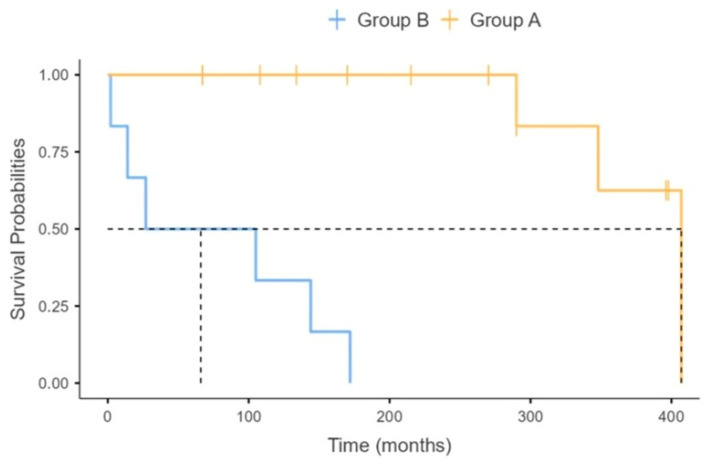
Kaplan–Maier estimates of tracheostomy-free survival after initiation of Long-Term Non-Invasive Ventilation, stratified according to the group of origin. Dotted line indicates median tracheostomy-free survival time.

The table shows that the proportion of patients who started LT-NIV post-acutely was significantly higher in group B with respect to the group A patients [3/6 vs. 0/12; *p*= 0.025]. Exact logistic regression according to multivariate analysis uncovered that there was a correlation at the limit of statistical significance (*p* = 0.051), between the unsatisfactory response to LT-NIV and the type of LT-NIV initiation, with an odds ratio of unfavorable outcome of 7.90 (95% CI, 0.99–123.2) for the patients who started the treatment following an episode of ARF. The median tracheostomy-free survival time was 171 (range: 2–407) months for all of the patients; it was significantly shorter in group B with respect to the group A patients [66 (range: 2–172) vs. 280 (range: 67–407) months; *p* = 0.010]. The tracheostomy-free survival time was 94.4 (95%CI, 84.4% to 100.0%) at 1 year, 83.3 (95%CI, 67.8% to 100.0%) at 3 years, and 83.3 (95%CI, 67.8% to 100.0%) at 5 years. Using the log-rank test, the stratification of patients according to the group of origin showed that the estimated tracheostomy-free survival was significantly reduced in group B with respect to the group A patients [66 (range: 14–NA) vs. 407 (range: 348–NA) months; *p* < 0.001] (Figure 1).

PFTs were only available for patients in group A during the follow-up period, showing an increase in FVC values equal to 74.9 ± 91.6 mL/yr.

There were 44 hospitalizations for the whole study group; these were linked to diagnoses of pneumonia (25), URTI (12), and aspiration pneumonia (7). The mean hospitalization rate, which was 0.09 (range: 0.00–1.44) episodes/yr for the whole study group, was significantly higher in group B with respect to the group A patients [0.35 (range: 0.06–1.44) vs. 0.07 (range: 0.00–0.18) episodes/yr; *p* = 0.007] (see Table 3).

## 4. Discussion

Although LT-NIV has been utilized in types 2 and 3 SMA patients since the beginning of the 1990s [9], data on its effect on patient-relevant outcomes are scarce and inconclusive. The current study retrospectively investigated a cohort of 18 types 2 and 3 SMA patients receiving home LT-NIV with the aim of evaluating a composite endpoint consisting of tracheostomy-free survival time and the need for hospitalization, two key factors affecting patients’ independence and QoL [19,20]. Our findings indicate that LT-NIV had a positive impact on the study participants: indeed, two thirds responded well, showing an estimated tracheostomy-free survival time of approximately 33 years, which is unquestionably longer with respect to that reported in heterogeneous groups of children and adults with progressive neuromuscular disorders (NMDs) [31,32]. The patients who responded well to treatment (group A) also showed a low number of hospitalizations linked to respiratory exacerbation (mean value: 0.07 episodes/yr), a finding that is in line with other studies reporting negligible respiratory morbidity in home care ventilator-dependent children with NMD [16,17,31].

While the response to LT-NIV was nevertheless predominantly satisfactory, one third of the patients (group B) experienced unfavorable outcomes and required the placement of a tracheostomy tube after only a brief period of NIV support and/or necessitated frequent hospitalizations due to respiratory exacerbation. A comparison of the two groups’ data uncovered that initiating LT-NIV in clinically unstable patients was indeed associated with an unfavorable outcome (OR = 7.90). Patout et al. likewise reported that when LT-NIV was initiated following an episode of ARF, it was found to be a poor prognostic feature in a heterogeneous population of patients attending two European expert centers [32].

An analysis of our results also uncovered that there was a trend toward higher, although still within the normal range, PaCO_2_ values at the time NIV was initiated in the group B patients [43.0 (range: 38.0–70.0) vs. 38 (range: 32.9–41.5) mmHg; *p* = 0.088]. As the majority of the non-ventilated types 2 and 3 SMA patients have daytime PaCO_2_ levels that are slightly lower than or fall into the lower end of the normal range [33], we hypothesize that even slightly higher PaCO_2_ levels could conceivably be a marker of advanced respiratory compromise and pending respiratory insufficiency. These results lead to the supposition that types 2 and 3 SMA patients with daytime PaCO_2_ levels in the upper normal range or slightly/moderately increased following an acute episode of respiratory exacerbation could be at risk of an unsatisfactory response to LT-NIV. It follows then that LT-NIV should ideally be offered to clinically stable patients whose daytime PaCO_2_ levels are between 36 and 38 mmHg. Accordingly, Veldhoen et al. reported that a level of PaCO_2_ approximating 38 mmHg could be considered a sign of pending respiratory insufficiency and an indication to initiate ventilatory support [33]. As our results confirm, a proactive care approach, prioritizing early recognition of pulmonary problems and frank, timely conversations about the decision to initiate LT-NIV, is of crucial importance. The significant proportion of NMD patients initiating LT-NIV after an acute exacerbation described by some studies [18,34] suggests that the tests currently used to identify, assess, and monitor respiratory function compromise are not entirely up to the task.

Pneumonia was the most common cause in our patients of ARF resulting in the need for ETI and, ultimately, tracheostomy. Other studies have likewise reported that respiratory tract infection (RTI) is the most frequent cause of acute exacerbation of chronic neuromuscular respiratory failure [35]. The finding suggests that clearly every effort should be made to prevent RTI in patients receiving home NIV. Non-professional care-givers should be provided information about the various measures of infection prevention, including proper cleaning and disinfection of the ventilator circuit, the importance of adequate hand hygiene, and the need for surgical masks during seasonal epidemics.

While 6 out of the 12 group A subjects were receiving Risdiplam, a disease-modifying therapy that improves motor function in types 2 and 3 SMA patients, none of the group B subjects were receiving this treatment. Although it is possible that Risdiplam might have contributed to preserving respiratory function and preventing exacerbations by maintaining respiratory muscle strength, it is unclear if an improvement in peripheral motor function would have led to clinically meaningful respiratory outcomes. Indeed, a recent systematic review and meta-analysis reported that the efficacy of Risdiplam on respiratory function in types 2 and 3 SMA children was inconsistent [36].

Our study has several limitations. First, this study’s design as a single-centered, retrospective, observational study is of course a limitation, but it is common knowledge that it would be impossible to design a prospective randomized trial for this patient population due to obvious ethical issues. Second, the study population was quite small, which is usually the case for clinical studies focusing on rare diseases and/or conditions. Third, this study’s time span was extremely long, but it is important to point out that no substantial changes were made in our ventilatory support protocols over that period of time. Fourth, the definition of treatment success was arbitrary. It was nonetheless based on available data on respiratory outcomes in types 2 and 3 SMA patients. Fifth, the diagnosis of SMA was formulated in four patients solely on the basis of their clinical presentation and electromyographic findings. Sixth and finally, subjective benefits of LT-NIV could not be evaluated in patients and/or care-givers, since QoL scales were not utilized. However, it is important to note that previous studies found a closed relationship between decreased number of hospitalizations and improved Health-Related Quality of Life in NMD patients receiving home mechanical ventilation [37]. This study’s most important strength is the fact that SMA was diagnosed in most cases following internationally accepted standards. Moreover, this study’s long duration permitted us to observe the effects of LT-NIV on these patients over a long period of time and to verify the efficacy and durability of the therapy.

To conclude, the current study confirms that LT-NIV has a positive impact on tracheostomy-free survival time and the need for hospitalization in types 2 and 3 SMA patients when it is initiated in clinically stable patients at an early stage of progressive ventilatory failure.

## Figures and Tables

**Table 1:** Patients’ baseline demographic, clinical, and pulmonary function characteristics at initiation of Long-Term Non-Invasive Ventilation. *p*-values refer to differences between groups A and B. (ACCI = Age-adjusted Charlson Comorbidity Index; BMI = body mass index; FVC = Forced Vital Capacity; NIV = Non-Invasive.)

	All Cases (n = 18)	Group A (n = 12)	Group B (n = 6)	*p*-Value
Age (years), median (range)	16.5 (2–55)	14.0 (2–45)	26.5 (12–55)	0.122
Gender (females/males), N	6/12	5/7	1/5	0.600
BMI (kg/m^2^), median (range)	15.8 (8.2–30.9)	14.4 (8.2–22.2)	20.7 (8.2–30.9)	0.345
Smokers, n (%)	2 (12.6)	1 (8.3)	1 (16.7)	0.542
SMA type (2/3)	10/8	8/4	2/4	0.321
RULM score, median (range)	7 (0–32)	8 (0–32)	7 (7–7)	0.999
Pts previously administered spinal fusion, N. (%)	5 (27.8)	4 (33.3)	1 (16.7)	0.999
Pts previously administered PEG, N. (%)	5 (27.8)	3 (25.0)	2 (33.3)	0.999
Pts with comorbidities, N. (%)	2 (11.1)	2 (16.6)	0 (0)	0.529
ACCI, median (range)	0 (0–1)	0 (0–1)	0 (0–0)	0.345
Pts with severe scoliosis, N. (%)	8 (44.4)	7 (58.3)	1 (16.6)	0.510
FVC, L median (range)	0.45 (0.15–3.34)	0.38 (0.18–3.34)	1.13 (0.15–3.03)	0.272
FVC, % median (range)	31 (8–81)	20 (8–81)	37 (10–71)	0.689
PaO_2_ (mmHg), median (range)	76.2 (67.0–88.2)	76.8 (67.0–88.2)	76.2 (73.0–82.0)	0.999
PaCO_2_ (mmHg), median (range)	39.2 (32.9–70.0)	38.0 (32.9–41.5)	43.0 (38.0–70.0)	0.088
Arterial pH, median (range)	7.42 (7.39–7.50)	7.42 (7.39–7.50)	7.41 (7.39–7.45)	0.563

**Table 2:** Prescription of SMN-enhancing treatment. *p*-values refer to differences between groups A and B.

	All Cases (n = 18)	Group A (n = 12)	Group B (n = 6)	*p*-Value
Nusinersen, n (%)	2 (11.1)	1 (8.3)	1 (16.6)	0.999
Risdiplam, n (%)				0.114
-First line treatment	3 (16.6)	3 (25.0)	0 (0)	
-Switch from Nusinersen	2 (11.1)	2 (16.6)	0 (0)	

**Table 3:** Initiation, complications, and clinical outcomes of Long-Term Non-Invasive Ventilation. *p*-values refer to differences between groups A and B. (LT-NIV = Long-Term Non-Invasive Ventilation.)

	All Cases (n = 18)	Group A (n = 12)	Group B (n = 6)	*p*-Value
LT-NIV initiation (elective/post-acute), n (%)	15/3	12/0	3/3	0.025
Daily NIV use at the end of follow-up, n (%)				0.303
• Nocturnal	4 (22.2)	4 (33.3)	0 (0)	
• Intermediate	8 (44.4)	4 (33.3)	4 (66.6)	
• Life support	6 (33.3)	4 (33.3)	2 (33.3)	
Complications, n (%)				0.245
• Nasal skin breakdown	2 (11.1)	1 (8.3)	1 (16.7)	
• Nasal congestion	1 (5.5)	0 (0)	1 (16.7)	
• Acute gastric distension	1 (5.5)	1 (8.3)	0 (0)	
Tracheostomy-free survival (months), median (range)	171 (2–407)	280 (67–407)	66 (2–172)	0.010
Hospitalization rate (episodes/yr), median (range)	0.09 (0.00–1.44)	0.07 (0.00–0.18)	0.35 (0.06–1.44)	0.007

## Data Availability

The clinical and respiratory function data that support the findings of this study are available at https://intranet.sanita.padova.it for further processing at the request of the interested party and were accessed on 1 April 2025. The data are not publicly available due to privacy restrictions.
